# Modeling of Measuring Transducers for Relay Protection Systems of Electrical Installations

**DOI:** 10.3390/s25020344

**Published:** 2025-01-09

**Authors:** Iliya Iliev, Andrey Kryukov, Konstantin Suslov, Nikolay Kodolov, Aleksandr Kryukov, Ivan Beloev, Yulia Valeeva

**Affiliations:** 1 Department of Heat, Hydraulics and Environmental Engineering, “Angel Kanchev” University of Ruse, 7017 Ruse, Bulgaria; iki@uni-ruse.bg; 2 Department of Transport Electric Power, Irkutsk State Transport University, 664074 Irkutsk, Russia; and_kryukov@mail.ru; 3 Department of Power Supply and Electrical Engineering, Irkutsk National Research Technical University, 664074 Irkutsk, Russia; appleforevor@mail.ru; 4 Department of Hydropower and Renewable Energy, National Research Technical University “Moscow Power Engineering Institute”, 111250 Moscow, Russia; 5 Department of Energy, Transbaikal State University, 672039 Chita, Russia; 6 Branch of JSC “System Operator of the Unified Energy System” Kuban Regional Dispatch Office, 350000 Krasnodar, Russia; k_kng@mail.ru; 7 Department of Transport, “Angel Kanchev” University of Ruse, 7017 Ruse, Bulgaria; ibeloev@uni-ruse.bg; 8 Department of Economics and Management, Russian University of Cooperation, 420034 Kazan, Russia; valis2000@mail.ru

**Keywords:** electric power systems, measuring transformers, symmetrical component filters, phase coordinates, modeling

## Abstract

The process of establishing relay protection and automation (RPA) settings for electric power systems (EPSs) entails complex calculations of operating modes. Traditionally, these calculations are based on symmetrical components, which require the building of equivalent circuits of various sequences. This approach can lead to errors both when identifying the operating modes and when modeling the RPA devices. Proper modeling of measuring transformers (MTs), symmetrical component filters (SCFs), and circuits connected to them effectively solves this problem, enabling the configuration of relay protection and automation systems. The methods of modeling the EPS in phase coordinates are proposed to simultaneously determine the operating modes of high-voltage networks and secondary circuits connected to the current and voltage transformers. The MT and SCF models are developed to concurrently identify the operating modes of secondary wiring circuits and calculate the power flow in the controlled EPS segments. This method is effective in addressing practical problems related to the configuration of the relay protection and automation systems. It can also be used when establishing cyber–physical power systems. For a comprehensive check of the adequacy of the MT models, 140 modes of the electric power system were determined which corresponded to time-varying traction loads. Based on the results of calculating the complexes of currents and voltages at the MT terminals, parametric identification of the power transmission line was performed. Based on this, the model of this transmission line was adjusted; repeated modeling was carried out, and errors were calculated. The modeling results showed a high accuracy when calculating the modules and phases of voltages using the identified model. The average error value for current modules was 0.6%, and for angles, it was 0.26°.

## 1. Introduction

The process of determining relay protection and automation settings involves complex calculations of forced and emergency conditions within the electric power systems (EPSs). Traditionally, they are performed using the method of symmetrical components, which requires the equivalent circuits of different sequences to be built for each of the scenarios considered. The results obtained are then used to determine the parameters of the relay protection and automation systems. This approach can result in errors during both the identification of the EPS operating conditions and the actual modeling of the relay protection and automation devices.

An alternative approach based on the development of a combined model of the EPS and relay protection and automation circuits offers numerous advantages. By utilizing phase coordinates, this approach enables the determination of currents, voltages, and power in low-voltage networks that power the relay protection and automation devices. The methods, algorithms, and the Fazonord software (ver. 5373-2024) based on [1] facilitate the modeling of any practically realizable AC systems with the inclusion of segments with different rated voltages in phase coordinates. The software rests on the original ideas of representing multi-wire components with mutually inductive and capacitive couplings in the form of lattice equivalent circuits (LECs). This approach makes it possible to consider transmission lines with a large number of wires and with any types of single-phase and three-phase transformers of various designs. The software contains models [2] of current and voltage transformers, which are energy sources in secondary wiring circuits. Therefore, calculation schemes can include relay protection and automation circuits. Based on such models, it is possible to consider the errors of these devices. When analyzing the operation of relay protection and automation systems, in addition to calculating any types of fault conditions, it is possible to model complex accidents in the EPS, for example, double ground faults, and address other urgent problems that arise in design and operation. These include the correct consideration of symmetrical component filters used in some relay protection schemes [3,4].

The significance of modeling the measuring transformers and symmetrical component filters is underscored by numerous publications focusing on the development and research of these devices. For example, the development of current transformer (CT) models for analyzing transient processes in relay protection and automation systems is the focus of [5]. Their study reveals that the presence of accurate CT models becomes necessary when assessing the behavior of relay protection and automation systems under operation. Particular attention is paid to address the nonlinearity related to the ferromagnetic core of the CT. The findings of a study on the CT frequency characteristics using the Jiles–Atherton model are presented in [6]. Their study shows that in the case of serious faults, the CT core can saturate, and the output current will not be linearly related to the measured one. Therefore, digital relays may not give a command to the switches to open, which will lead to dangerous or destructive consequences. A compensation method using the Jiles–Atherton model is proposed to reproduce the primary current from the measured secondary current during transient processes. The phenomenon of complete reversibility of magnetization is considered. The effect of direct current (DC) bias on the CT characteristics is analyzed in [7]. DC transmission lines, electrified transport, and geomagnetic disturbances can change the CT characteristics, which affect the accuracy of the relay protection and automation systems. The effect of these factors on the current transformer is analyzed. The simulation results show that during the normal operation of CT, when a small direct current IDC flows through its primary winding, the CT characteristics change little. With significant IDC, however, the CT core saturates, and noticeable harmonic distortions occur. Materials on the design and manufacture of high-frequency CTs (HF CTs) are given in [8]. Their study reveals that HF CTs are well suited as sensors for recording signals on power cables, such as partial discharges. If the HF CT bandwidth aligns with the corresponding indicator of the measured parameter, high sensitivity can be achieved. Their study indicates that the optimization of the HF CT is a challenging task, since various design features affect the transfer function. The proposed HF CT model can serve as an effective tool for modeling and optimizing the sensor. The optimal design of the differential zero-sequence (ZS) current transformer is described in [9]. Their study shows that in networks with low grounding resistance, the ZS CT may fail to provide sufficient accuracy. This problem can be tackled by using a flux-constrained ZS CT. An optimal design method for such a device is developed, and its operating principle is presented. A winding method and an algorithm for optimizing the air gap of the ZS CT are considered to minimize errors. The presented modeling and experimental results show that the proposed method can be used to design a high-precision flux-constrained ZS CT. The studies of CT characteristics using an improved hysteresis model based on the Brillouin function are described in [10]. In complicated CT operation conditions, which cannot be explored experimentally, the development of an accurate CT model is essential to cope with electromagnetic problems. The typical CT model is usually based on the Langevin function. However, some solutions obtained with this basis need to be adjusted. The authors apply the Brillouin function and build a numerical model of the CT. It contains only the solutions that are consistent with the experiments and is more practical and capable of accurately reflecting the real characteristics of the CT. The operation of the current transformer under transient conditions is considered in [11]. Their paper presents a detailed analysis of the CT. The parameters of the equivalent circuit are determined using the finite element method and verified with measurements. A mathematical model of the CT with a nonlinear magnetization curve is used to conduct the study under normal and faulty conditions. The enhancement of the CT accuracy through harmonic distortion compensation is considered in [12]. Their study indicates that CTs have problems due to the magnetization characteristics of the cores. Although, the saturation of the cores can occur due to high currents, there is weak nonlinearity during normal operation as well. A typical spectrum consists of a fundamental component and harmonics, which have significantly smaller amplitudes. Their study proposes a method to compensate the harmonics, using calculations to estimate its performance. The current transformer model is tested using the EMTP-ATP software in [13]. Several models developed for studying electromagnetic transient processes are discussed, as well as tests performed for validation. Particular attention is paid to the magnetization branch, which proved to be a critical parameter of the CT. The saturation of high-frequency CTs used to measure partial discharges (PDs) or other current pulses on power cables is examined in [14]. In addition to the PD signal, a high current with a frequency of 50 or 60 Hz is usually transmitted. They are superimposed and inductively coupled with the HF CT sensor. The magnetic field of the 50 or 60 Hz current is strong enough to saturate the core, which distorts the measurement results, reduces the sensitivity of the HF CT, and causes unwanted output voltage peaks. To avoid this undesired effect, air gaps are used, but this leads to a decrease in the ability to detect PD. This problem can be tackled using frequency-dependent impedance at the HF CT output. The findings indicate that it can be used without air gaps in the core. A technique for thermal modeling of an oil-immersed CT is presented in [15]. Their study reveals that the service life of a CT is determined by the hottest point temperature (HPT). An experimental setup is developed to measure the HPT. To this end, thermocouples and fiber optic sensors are built into the secondary winding. The collected data are used in combination with thermal models. The predicted temperature profiles based on these parameters are in good agreement with the measured values and can be used for autonomous monitoring under overload conditions. The CT modeling for measuring fast power module current is discussed in [16]. The results of a successful application of CT manufactured using silicon steel laminations are presented. The CT performance is competitive with existing devices due to its wide bandwidth, small size, simple design, galvanic isolation, and low cost. The authors present a detailed theoretical analysis and design recommendations. In addition, the errors associated with the influence of the main geometric parameters are determined. A prototype current transformer was manufactured and tested to validate the theoretical analysis. The experimental results are in good agreement with the theory developed by the authors. An algorithm for CT modeling using magnetization curve fitting through optimization is described in [17]. Their paper introduces a new CT model, which enables the determination of the secondary current waveform while taking saturation into account. Understanding the magnetic core behavior is an important criterion for CT design. Three models are proposed, including the Von Bertalanffy model, the Frohlich model, and a generic polynomial model, which aid in simulating the magnetization characteristics of various magnetic materials. The parameters are calculated using optimization algorithms. A CT sample is experimentally tested to validate the model. Methods for modeling CTs under dynamic conditions are presented in [18]. Most of the studies examining the current and voltage transformers are aimed at calculating their characteristics in steady states to determine the compliance of devices with metrological requirements. Such studies often employ ladder equivalent circuits or rely on field calculations, enabling a more accurate measurement of magnetic flux distribution. The nonlinearity of the magnetization curve necessitates the approaches that incorporate both a model of the device itself and the circuits connected to it. In particular, of practical interest is the analysis of the stability of a voltage transformer (VT) against the ferroresonance phenomena. A model of the CT thermal circuit is developed to calculate the hottest spot temperature in [19]. This model takes into account meteorological parameters, including wind speed, among others. It encompasses steady-state and dynamic thermal fields. The modeling results and experimental data agree well, demonstrating that the deviation does not exceed 10%, thereby validating the accuracy. The harmonics of currents are measured using a current transformer in [20]. The analysis of CTs when excited by non-sinusoidal currents is performed using a nonlinear model that considers the hysteresis loop. It is concluded that CTs with a class rating of 0.6 or higher deliver sufficiently precise measurements of current harmonics, but inaccuracies in the phase angle can lead to unacceptable errors. The analysis of how digital elements (DEs) of relay protection function during CT saturation is presented in [21]. The consideration is given to the digital element algorithms that rely on calculations based on instantaneous values and use Fourier filters. The influence of factors such as sampling time and the damping decrement of the aperiodic component of the input signal on the accuracy of the digital element operation is investigated. The modeling results are validated. The study reveals that the nature of the error change is different for the algorithms using instantaneous values and Fourier filters. With an increase in the damping decrement, the absolute errors decrease. A method of iterative calculation of CTs using an approximated magnetization curve is proposed in [22]. The most common downsides of models of electromagnetic processes in CTs are analyzed. Following the results obtained, simpler and more practical algorithms are developed. They make it possible to take into account the residual magnetic induction and determine all the necessary characteristics of CTs. The simulation results for an optical CT using operational amplifiers are delivered in [23]. It is noted that ferromagnetic CTs are widely used in energy systems. However, numerous problems related to starting currents, saturation, transformation ratios, and others restrict their application, which is why optical CTs (OCTs) are proposed. OCTs can resolve almost all the problems encountered by traditionally designed CTs. The technology of detecting current transformer saturation and compensation correction based on an intelligent algorithm is described in [24]. Their study shows that with the development of DC power transmission lines, the influence of magnetic bias becomes increasingly serious. The discrete wavelet transform and the random forest model are employed to assess the effect of the fault current on the CTs and to implement the correction of distortions. The study by [25] determines the accuracy of harmonic measurement using CTs. They found that CT errors depend on the accuracy class. For low-power CTs, the fixed error limits correspond to the harmonic components. Their paper presents a method for modeling the harmonic ratio and phase errors. It also proposes an alternative approach to specifying accuracy requirements at a given harmonic, which should depend on its relative value with respect to the fundamental. An algorithm for modeling a CT operating under thermal stress is introduced in [26]. Their study primarily focuses on magnetic materials under Curie temperature. The Jiles–Atherton and flux tube models are used to reproduce static and dynamic hysteresis loops. Six parameters are optimized for each temperature. The measurement of the nonsinusoidality factor of a single-phase voltage utilizing symmetrical component filters is examined in [27]. There are also a number of publications on transformer modeling [28,29,30,31].

The analysis of the publications discussed above reveals that they consider many important aspects of modeling the transformers, which establishes a methodological framework for developing new approaches in this area. The modern electric power industry is undergoing a transformation through the intelligent automation of control processes. The purpose of this transformation is to ensure that electric power systems have rational behaviors, which implies the ability to track changes in the environment and, if necessary, adapt and reconfigure themselves accordingly. To achieve this, the concept of smart grids is used. On this basis, it is planned to build a highly reliable, automatically balancing, and self-controlled EPS. Such systems will be able to receive electricity from various sources (including non-conventional ones) with minimal personnel involvement. Smart grid technologies imply the widespread use of advanced sensor, communication, and control components, which will enhance the levels of reliability and safety. To implement the smart grid concept, it is necessary to boost the efficiency of modern relay protection and automation devices, which are based on microprocessor tools and allow the implementation of advanced algorithms for controlling emergency conditions. However, the existing practice of configuring relay protection and automation systems relies on traditional methods, which involve identifying the emergency conditions using the symmetrical components method. Such approaches are not efficient enough and, in addition, when applied, can result in significant inaccuracies due to failure to take into account the errors of CTs and VTs, as well as the operating features of the EPS.

An original approach is proposed below to configure relay protection and automation devices, which is centered for the development of comprehensive EPS models. They include measuring transformers (CTs and VTs) and symmetrical component filters. There are several differences between this approach and traditional methods; the EPS models are designed using phase coordinates, which allow for the identification of operating conditions for any type of multiple longitudinal and transverse imbalances; comprehensive modeling of the power grid and secondary circuits takes into account real measurement errors.

## 2. Modeling of Measuring Transformers in Phase Coordinates

**Figure 1:**
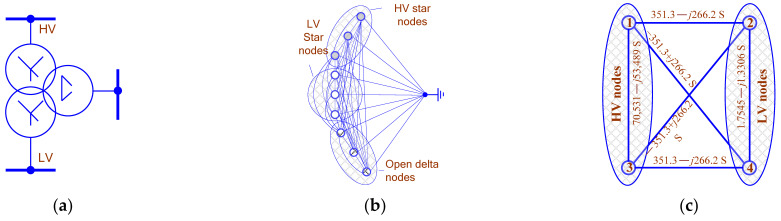
Circuits of voltage transformers (VTs) and current transformers (CTs): (**a**)—VT circuit; (**b**)—VT LEC; (**c**)—CT LEC; HV—high voltage; LV—low voltage.

Based on phase coordinates, it is possible to implement the models of EPS power components, such as transmission lines and transformers, allowing for the determination of highly unbalanced conditions. Most often, such conditions arise in electrical networks adjacent to traction substations (TSs) of main railways [1]. One of the most effective approaches to the implementation of these models is the use of lattice equivalent circuits (LECs), which are a set of RLC circuits. LECs (Figure 1b,c) have a fully connected topology, i.e.,

$$LEC:NOD\cup BRANC,\forall i,k\subset NOD\to branc_{i,k}\subset BRANC$$

where NOD and BRANC are sets of nodes and branches, respectively.

Modeling of measuring VTs can rely on the approach similar to that used for power devices. In this case, the LEC matrix is formed on the basis of the following relationship.

(1) $${\underline{Y}}_{PC}=\left[\begin{array}{cc} -{\underline{Z}}_{E}^{-1} & {\underline{Z}}_{E}^{-1}\\ {\underline{Z}}_{E}^{-1} & -{\underline{Z}}_{E}^{-1} \end{array}\right]$$

where ${\underline{Z}}_{E}=\underline{Z}-j\omega W_{1}{\underline{R}}_{M}^{-1}W_{2}$; $\underline{Z}$ is the matrix corresponding to the electrical resistances of the Windings; ${\underline{R}}_{M}$ is the magnetic parameter matrix; $W_{1}$ and $W_{2}$ are matrices composed of the numbers of turns of the windings [1]. Figure 1a shows the circuit of the voltage transformer. The graphs of the VT LEC and CT LEC are shown in Figure 1b,c.

**Figure 2:**
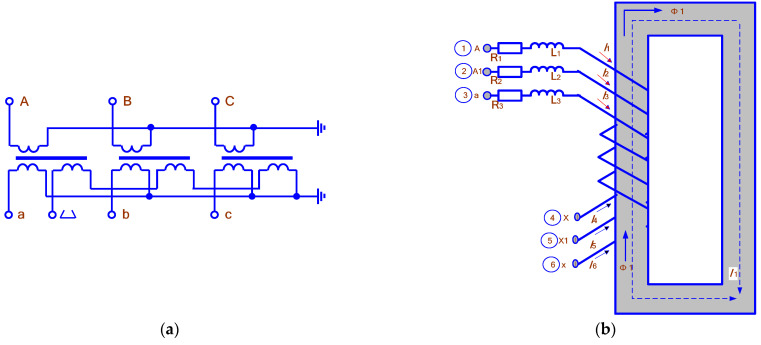
Circuit of the voltage transformer (**a**) and its model (**b**).

Figure 2 shows the Circuit of the voltage transformer and its model.

Electromagnetic processes in a transformer are described by the following system of equations:

(2) $$\left(R_{1}+j\omega L_{1}\right){\dot{I}}_{1}+j\omega w_{1}{\dot{\Phi }}_{1}={\dot{U}}_{1}$$

(3) $$\left(R_{n}+j\omega L_{n}\right){\dot{I}}_{n}+j\omega w_{n}{\dot{\Phi }}_{1}={\dot{U}}_{n}$$

(4) $${\dot{I}}_{1}w_{1}+{\dot{I}}_{2}w_{2}+..+{\dot{I}}_{n}w_{n}-{\underline{R}}_{m}{\dot{\Phi }}_{1}=0$$

(5) $$w_{i}=\frac{U_{i}\sqrt{2}}{\omega B_{2m}S_{1}}=\frac{4502U_{i}}{B_{2m}S_{1}}$$

(6) $${\dot{H}}_{1}l_{1}=\frac{{\dot{B}}_{1}l_{1}}{\mu_{0}{\underline{\mu }}_{r}}={\underline{R}}_{m}{\dot{\Phi }}_{1}$$

(7) $${\underline{R}}_{m}=\frac{l_{1}}{\mu_{0}({{\underline{\mu }}_{r}}^{\prime }-j{{\underline{\mu }}_{r}}^{\prime \prime })S_{1}}$$

(8) $${{\underline{\mu }}_{r}}^{\prime }-j{{\underline{\mu }}_{r}}^{\prime \prime }=\frac{l_{1}({{\underline{R}}_{m}}^{\prime }-j{{\underline{R}}_{m}}^{\prime \prime })}{\mu_{0}({{\underline{R}}_{m}}^{\prime 2}+{{\underline{R}}_{m}}^{\prime \prime 2})S_{1}}$$

where ${\underline{R}}_{m}={R_{m}}^{\prime }+j{R_{m}}^{\prime \prime }$ is the magnetic resistance of the core.

This system is defined by a matrix that can be represented as follows:

(9) $$\left|\begin{array}{ccccc} \overset{\cdot }{I_{1}} & \overset{\cdot }{I_{2}} & \ldots & \overset{\cdot }{I_{n}} & \overset{\cdot }{\Phi }\\ R_{1} & 0 & \ldots & 0 & j\omega w_{1}\\ 0 & R_{2}+j\omega L_{2} & \ldots & 0 & j\omega w_{2}\\ \ldots & \ldots & \ldots & \ldots & \ldots \\ 0 & 0 & \ldots & R_{n}+j\omega L_{n} & j\omega w_{n}\\ w_{1} & w_{2} & \ldots & w_{n} & -R_{m} \end{array}\right|$$

The VT is characterized by significant differences in the voltages of the primary and secondary windings, which lead to large leakage inductances. Therefore, the open-circuit (no-load) equations must take into account the voltage drop across the impedance $R_{1}+j\omega L_{1}$:

(10) $$\left(R_{1}+jX_{1}\right){\dot{I}}_{1x}+j\omega w_{1}{\dot{\Phi }}_{1}={\dot{U}}_{1}$$

(11) $${\dot{\Phi }}_{1}=\frac{{\dot{I}}_{1x}w_{1}}{{\underline{R}}_{m}}$$

(12) $$I_{1x}=\frac{i_{x}S_{H}}{U_{1}}$$

(13) $$\left(R_{1}+jX_{1}+j\frac{\omega {w_{1}}^{2}}{{\underline{R}}_{m}}\right){\dot{I}}_{1x}={\dot{U}}_{1}$$

After multiplying by the conjugate complex of the current, we can write the equation as follows:

(14) $$\left(R_{1}+jX_{1}+j\frac{\omega {w_{1}}^{2}}{{\underline{R}}_{m}}\right){I_{1x}}^{2}=P_{x}+jQ_{x}$$

(15) $$Q_{x}=\sqrt{{\left(i_{x}S_{H}\right)}^{2}-{P_{x}}^{2}}$$

(16) $$R_{x}+jX_{x}=\frac{P_{x}+jQ_{x}}{{I_{1x}}^{2}}$$

(17) $${\underline{R}}_{m}=\frac{j\omega {w_{1}}^{2}}{R_{x}-R_{1}+j\left(X_{x}-X_{1}\right)}$$

Magnetic resistance is calculated after determining the short circuit parameters using the method described in [1].

**Figure 3:**
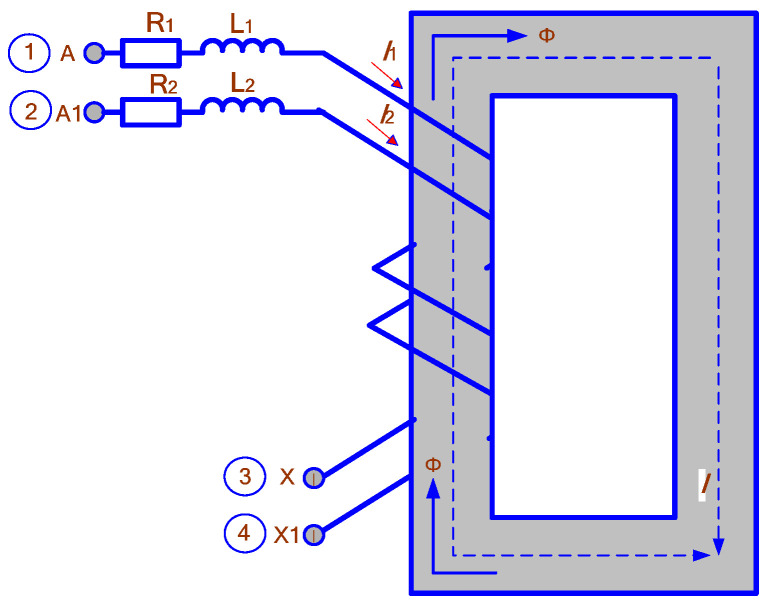
CT model circuit.

The CT model is built using the circuit shown in Figure 3.

The power transformer model described in [1] can be used for CT by transforming its parameters following the algorithm that includes the following seven steps:

1. Input the following initial data for modeling: $S_{n}$ is the nominal power of the secondary winding of CT; ρ is the specific resistance of the winding material; $l_{w}$ is the length of the average turn of the secondary winding, and cross-section $F_{2}$ is the wire the winding is made of; the number of turns of primary $w_{1}$ and secondary $w_{2}$ windings; dimensions of the CT magnetic core; its cross-section area *F*_1_; length *l* of an average magnetic line of force; CT current $f_{I}$ and angular δ errors;

2. Calculate the resistances corresponding to the nominal load of CT as follows:

(18) $${\underline{Z}}_{2n}=R_{2n}+jX_{2n}=\frac{S_{n}}{I_{2n}^{2}}\left(\cos\varphi +j\sin\varphi \right)$$

(19) $$R_{2}=\frac{\rho l_{w}w_{2}}{F_{2}}$$

(20) $$X_{2}=4\pi \cdot 10^{-7}f{w_{2}}^{2}h_{i}\ln\frac{D_{out}}{D_{in}}$$

where $h_{i}$ is the height of the magnetic core factoring in secondary winding and insulation, determined in most of the practical cases as the thickness of toroid located horizontally, m; $D_{out}$ and $D_{in}$ are the outer and inner diameters of the magnetic core, considering the secondary winding and insulation, m;

3. Determine the primary current and power characterizing of the equivalent power transformer using the formulas:

(21) $${\dot{I}}_{1n}=-I_{2n}\frac{w_{2}}{w_{1}}\left(1+f_{I}-j\delta \right)$$

(22) $${\dot{S}}_{1n}={I_{2n}}^{2}\left\{R_{2}\left(1+f_{I}\right)+R_{2S}+X_{2}\delta +j\left[X_{2}\left(1+f_{I}\right)-R_{2}\delta +X_{2S}\right]\right\}\left(1+f_{I}+j\delta \right)$$

where $I_{2n}$ is the nominal current of the secondary winding; $R_{2S}=R_{2}+R_{2n}$ and $X_{2S}=X_{2}+X_{2n}$; $R_{2n}=\frac{S_{n}\cos\phi }{I_{2n}^{2}}$; $X_{2n}=\frac{S_{n}\sin\phi }{I_{2n}^{2}}$;

4. Calculate the magnetic flux corresponding to the nominal operating conditions and determine the voltages of the CT windings that will be used in the model:

(23) $${\dot{\Phi }}_{n}=I_{2n}\frac{R_{2S}+jX_{2S}}{-j\omega w_{2}}$$

(24) $$U_{1w}=\omega w_{1}\Phi_{n}$$

(25) $$U_{2w}=\omega w_{2}\Phi_{n}$$

5. Calculate the parameters characterizing the short-circuit conditions of the equivalent power transformer:

(26) $$u_{k}=100\cdot \sqrt{\frac{A^{2}+B^{2}}{C^{2}+D^{2}}}$$

where $A=\left[\left(2+f_{I}\right)R_{1}+\delta X_{1}\right]$; $B=\left[(2+f_{I})X_{1}-\delta R_{1}\right]$; $C=\left[\left(2+f_{I}\right)R_{1}+\delta X_{1}+\frac{{w_{1}}^{2}}{{w_{2}}^{2}}R_{2n}\right]$; $D=\left[\left(2+f_{I}\right)X_{1}-\delta R_{1}+\frac{{w_{1}}^{2}}{{w_{2}}^{2}}X_{2n}\right]$.

(27) $${\dot{S}}_{1k}={I_{2n}}^{2}\left\{R_{2}\left(1+f_{I}\right)+R_{2}+X_{2}\delta +j\left[X_{2}\left(1+f_{I}\right)-R_{2}\delta +X_{2}\right]\right\}\left(1+f_{I}+j\delta \right)$$

where $R_{1}=R_{2}\frac{{w_{1}}^{2}}{{w_{2}}^{2}}$ and $X_{1}=X_{2}\frac{{w_{1}}^{2}}{{w_{2}}^{2}}$.

6. Precalculate the amplitude of the nominal magnetic induction of the CT using the following expression:

(28) $$B_{m}=\frac{4502U_{2w}}{w_{2}F_{1}}$$

where *U*_2*w*_ is given in kilovolts.

The obtained value $B_{m}$ must be adjusted; the numbers of turns $w_{1}$ and $w_{2}$ must be close to those set in point 1;

7. Determine the current and power corresponding to the no-load conditions.

(29) $${\dot{I}}_{1h}=-I_{2n}\frac{w_{2}}{w_{1}}\frac{\left(R_{2n}+jX_{2n}\right)\left(f_{I}-j\delta \right)}{R_{2S}+jX_{2S}}$$

(30) $$i_{x}=\frac{I_{1h}}{I_{1n}}\cdot 100\%$$

(31) $${\dot{S}}_{1h}=P_{h}+jQ_{h}=\frac{{w_{2}}^{2}}{{w_{1}}^{2}}{I_{2n}}^{2}\frac{{\left(R_{2n}+jX_{2n}\right)}^{2}}{R_{2S}+jX_{2S}}\left[\frac{{w_{1}}^{2}}{{w_{2}}^{2}}\left(f_{I}-j\delta \right)-\frac{j2\delta \left(R_{1}+jX_{1}\right)}{R_{2S}+jX_{2S}}\right]$$

After determining *i_x_* and *U*_2*w*_, adjustments may be required to ensure the required CT errors are fixed.

**Figure 4:**
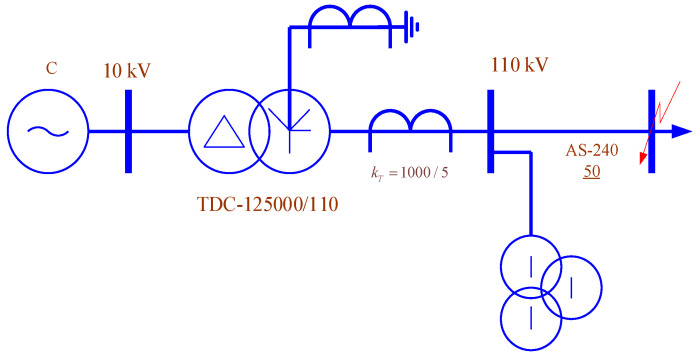
Original electrical circuit.

The adequacy of the described CT modeling methods is validated by calculating the emergency parameters of the network, whose circuit is shown in Figure 4. RL circuits with impedances of 0.64 + j0.48 Ohm were used as CT loads in this circuit. The resistance of the grounding device (Figure 4) was taken to be 0.33 Ohm. The currents and voltages of single-phase short circuits determined at the receiving end of the 110 kV transmission line are summarized in Table 1. Analytical calculations of the short-circuit conditions were carried out using the method of symmetrical components. The obtained currents were recalculated for the secondary circuit of the CT using the nominal transformation ratio. As a result, a current of 7.07 A was obtained, differing from the data in Table 1 by 0.57%.

The results obtained indicate that the use of phase coordinates and LEC allows for the adequate modeling of measuring transformers.

**Figure 5:**
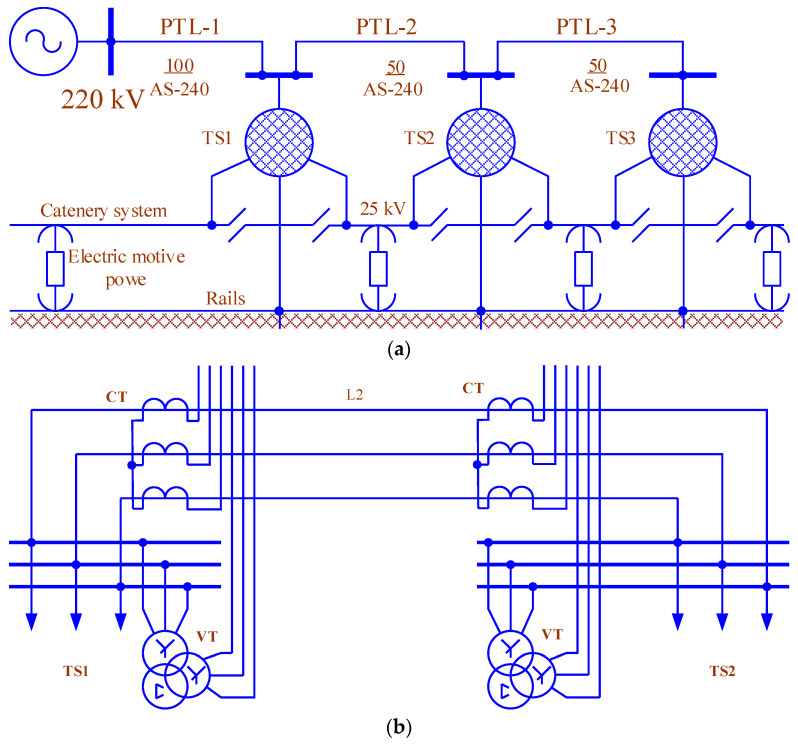
The 220 kV electrical networks supplying traction substations of the main railway (**a**) and the CT and VT connection circuit (**b**).

The joint consideration of high-voltage electrical networks and secondary switching circuits that feed the relay protection and automation devices is exemplified by the circuit presented in Figure 5a. The modeling was performed using the Fazonord software [1]. The calculation circuit included models of three 220 kV transmission lines made of AC-240 wires, the same number of TDTNZh-40000/230/27.5 traction transformers (Russia), and a traction network of double-track sections. In addition to them, the models of CTs and VTs were used. Their connection to the 220 kV line is shown in Figure 5b. The track profile features two lengthy climbs in the first inter-substation area, creating significant traction loads when down trains pass. The schedule of three such trains with masses of 5000 tons was used.

**Figure 6:**
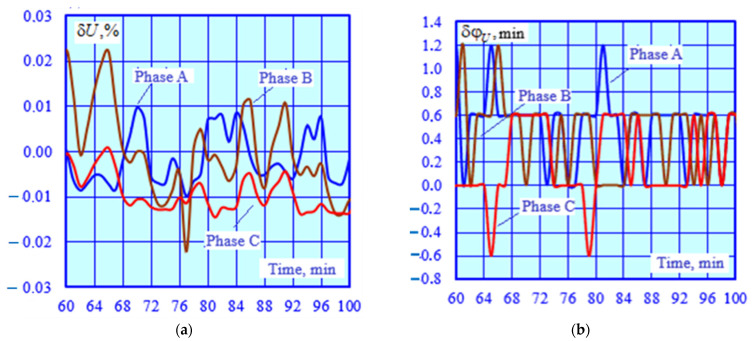
Errors in voltage magnitudes (**a**) and phases (**b**).

**Figure 7:**
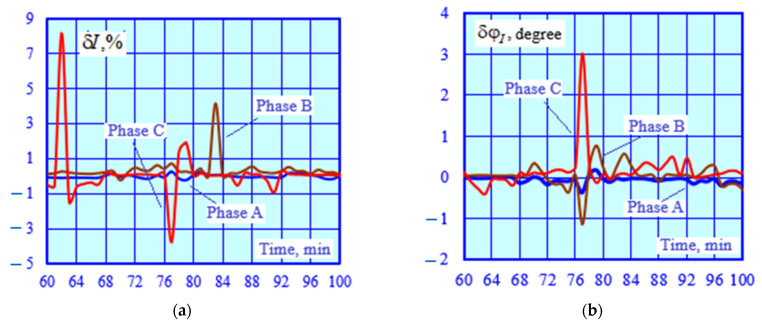
Errors in current magnitudes (**a**) and phases (**b**).

The adequacy of the CT and VT models was checked by identifying a series of EPS operating modes corresponding to time-varying traction loads. A total of 140 operating modes were calculated. Using the current and voltage phasors calculated at the CT and VT terminals, a parametric identification of PTL 2 was carried out using the method described in [32]. The results of the identification were utilized to adjust the model of this power transmission line; the modeling was repeated, and the errors were calculated (Figure 6 and Figure 7).

The graphs shown in Figure 6 demonstrate a high accuracy of calculating voltage magnitudes and phases using the identified transmission line model. The abnormal error spikes depicted in Figure 7 are linked to small primary CT currents. Excluding these spikes, the average value of the current magnitude error is 0.6%, while the angular error stands at 0.26%°.

## 3. Modeling of Symmetrical Component Filters

Faults in three-phase EPSs are identified using voltage and current symmetrical component filters (SCFs), which allow for the detection of components of positive, negative, or zero sequences. Below is a brief description of the most common SCF schemes.

The negative sequence voltage filter (NSVF) is built on the basis of RC phase-shifting circuits connected to the VT secondary winding. With certain parameters of the circuits, the voltage at the NSVF output is zero if the input voltage does not contain negative sequence components. The positive sequence voltage filter (PSVF) can be implemented on the basis of the same scheme if the connection of any pair of phases is swapped. The zero sequence voltage filter (ZSVF) is implemented by serially connecting the phases of the VT secondary windings. In particular, the NTMI type VT is made with five rods and three windings with the third winding connected in an open delta arrangement.

The negative sequence current filter (NSCF) is equipped with a transreactor designed to obtain an EMF on the secondary winding shifted by 90° relative to the difference in currents of the two primary windings, a compensation transformer, an adjustable resistor, and an output low-impedance relay. The transreactor can be adjusted to obtain the required value of mutual induction resistance. The voltage of its output winding, shifted by 120° and proportional to the difference in currents of phases A and B, is added to the voltage drop across the resistor due to the current of phase C. As a result, the total voltage on the winding of the low-impedance output relay equals zero when the input currents do not contain negative sequence components. The difference in phase currents is used to tune out the zero sequence current. The zero sequence current filter (ZSCF) can be constructed by summating either phase currents or magnetic fluxes created in the core of the ZSCT.

The diagrams of the SCF models implemented in the Fazonord software are presented in Table 2. A detailed description of these models is given in [4].

**Figure 8:**
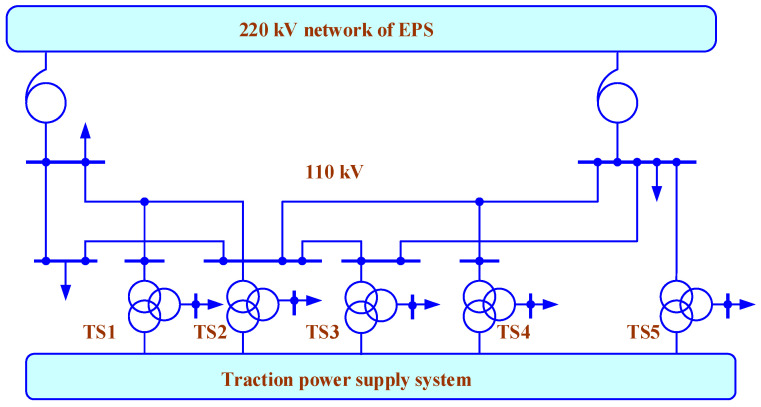
Diagrams of electrical network.

**Figure 9:**
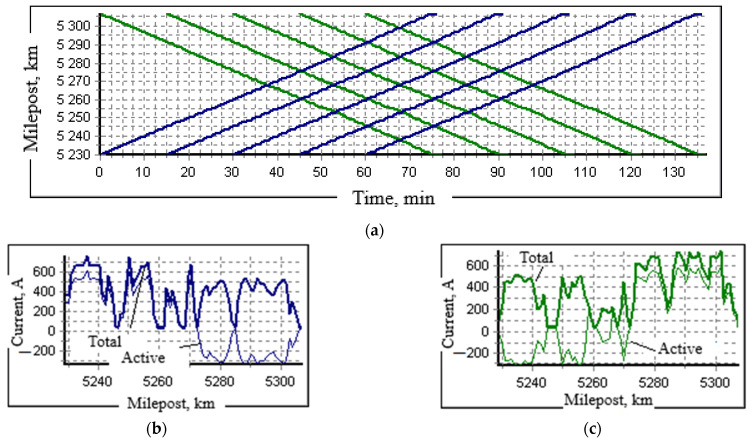
Schedule of trains weighing 4000 tons (**a**) and traction current profiles for up (**b**) and down (**c**) trains.

The possibilities of using the developed models are illustrated by identifying the operating modes of the power supply system of a railway section incorporating a 25 kV traction network. The circuit of the high-voltage part of the network is shown in Figure 8. It presents five substations: TS 1–TS 5. The model block corresponding to the traction power supply system includes four inter-substation areas with a double track with a 10 kV line made of A-35 wires located on the supports of the catenary system. The section is powered by a 110 kV line and a 220 kV transmission line parallel to it. In the diagram, the traction substation TS 2 is represented by a model of a TDTNZh-40000 transformer (Russia) with a 10 kV winding connected in a star with a grounded neutral to eliminate the effects of the capacitive influence of the short circuit. It feeds the longitudinal power supply line TS 2–TS 3, at the end of which the TM-25-10/0.4 transformer (Russia) with a balanced load of 6 + j4 kVA per phase is connected. Two HTMI-10 type VTs and two positive and negative sequence voltage SCFs are connected to the 10 kV winding of TS2. The train schedule and traction current profiles are shown in Figure 9.

**Figure 10:**
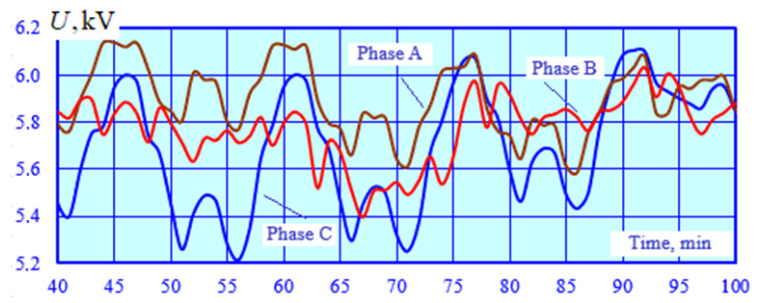
Change in phase voltages at the starting end of longitudinal power supply line.

**Figure 11:**
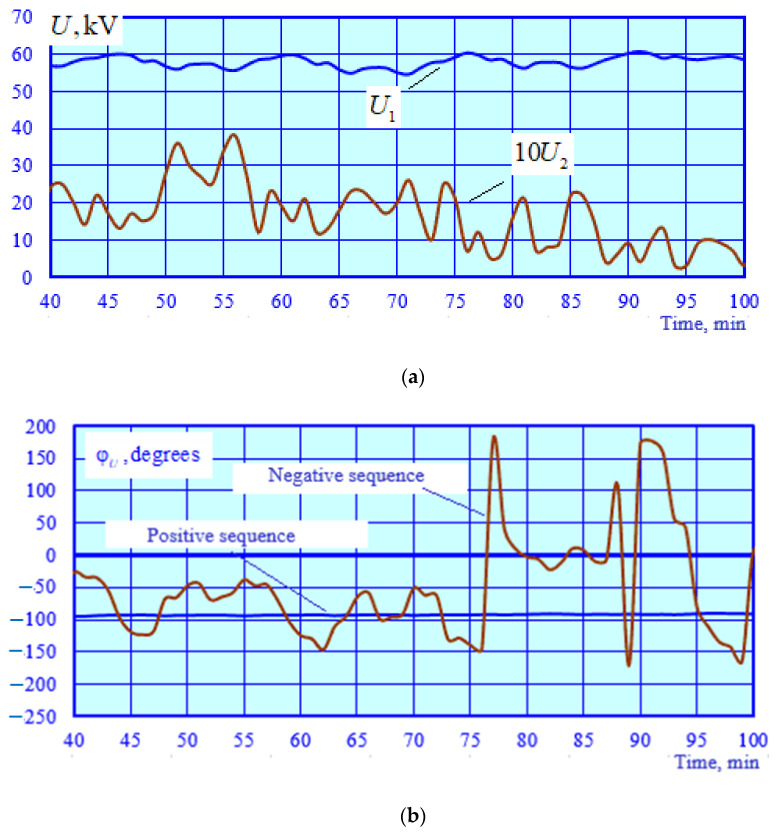
Change in magnitudes (**a**) and phases (**b**) of positive and negative sequence voltages at the F1 and F2 outputs; the magnitude of the negative sequence voltage is increased tenfold for visual clarity.

**Figure 12:**
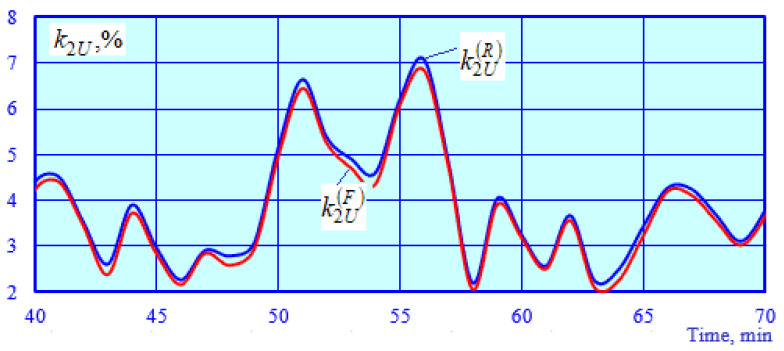
Changes over time in the output voltage of the NSVF and the unbalance factor of the 10 kV busbars of TS 2; $k_{2U}^{\left(F\right)}$ is obtained using the PSVF and NSVF models; $k_{2U}^{\left(R\right)}=\frac{100U_{2}}{U_{1}}$; *U*_1_ is the positive sequence voltage; *U*_2_ is negative sequence voltage.

**Figure 13:**
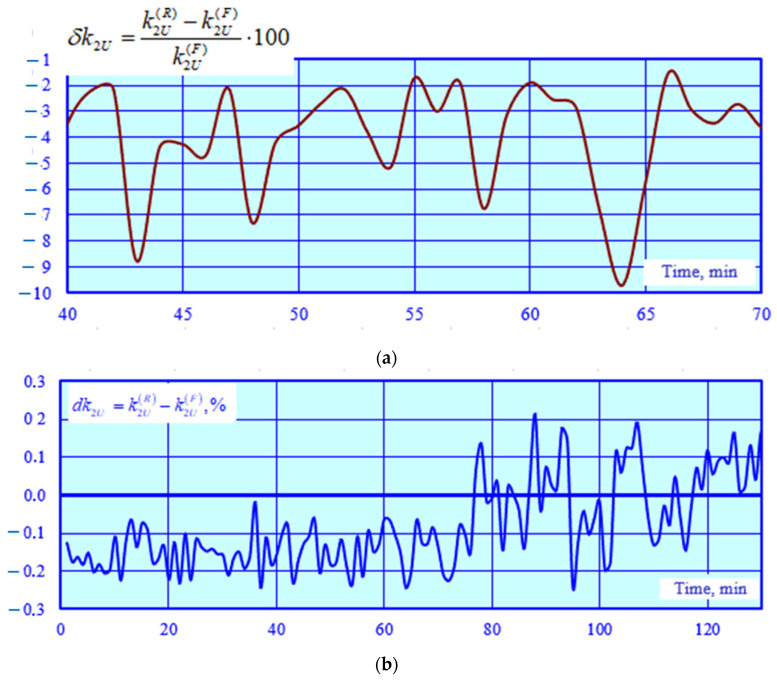
Graphs of relative (**a**) and absolute (**b**) errors in determining the negative sequence unbalance factor.

Modeling results for the operating modes of the power supply system are presented in Figure 10, Figure 11, Figure 12 and Figure 13.

Figure 10 shows the graphs of the time dependencies for the voltage at the starting end of the longitudinal power supply line. Significant variations in the PTL voltage are observed due to a marked change in traction loads and the electromagnetic interference caused by the catenary system. Figure 11 illustrates the graphs of time-varying negative sequence unbalance factor values calculated and obtained using the SCF. Figure 12 demonstrates the time dependencies of the voltage at the SCF terminals. Figure 13 shows the graphs of the absolute and relative errors in determining the unbalance factor *k*_2*U*_.

The dependency $k_{2U}=k_{2U}\left(t\right)$ for 10 kV busbars differs from the voltage ratio by an average of 4.7%, with the exception of very small values, at which the errors of the SCF have an effect.

The developed SCF models, as well as the CT and VT models will enhance the accuracy of the operating mode calculations, the results of which are used in configuring and diagnosing relay protection and automation devices.

The concept of cyber–physical systems (CPSs) [33] is currently playing a pivotal role in the implementation of smart grid technologies, serving as a basis for the establishment of advanced, efficient, and reliable electric power systems. This involves a comprehensive integration of digital components designed to process large amounts of information coming from sensors and, on this basis, model physical objects and control them. For the CPS to operate effectively, it is essential to develop robust digital models that can be implemented using the aforementioned methods.

## 4. Conclusions

To implement intelligent electrical networks and cyber-physical power supply systems, it is necessary to solve the problems of increasing the efficiency of relay protection and automation devices, since when using traditional methods of setting up relay protection and automation, significant inaccuracies may arise due to failure to take into account the errors of current transformers and voltage transformers.

The presented models of current and voltage transformers allow for the determining of the modes of secondary switching circuits together with calculations of the flow distribution of controlled power networks. The conducted modeling showed that this approach provides a high accuracy of modeling emergency requirements. The average error value for current modules was 0.6%, and for angles, it was 0.26°.

Models of filters of symmetric components have been developed, which allow us to isolate components of direct, reverse, and zero sequences. They are widely used in modern power direction relay protection and protection against currents and voltages of reverse sequence.

The methodology presented in this article and the digital models developed on its basis are applicable for solving practical problems related to the setting up of relay protection and automation devices. In addition, the described approach can be used to create cyber–physical EPS and power supply systems.

## Figures and Tables

**Table 1:** Short-circuit calculation results.

Parameter	Measurement Units	Short-Circuit Type								
		$K_{A}^{\left(1\right)}$			$K_{B}^{\left(1\right)}$			$K_{C}^{\left(1\right)}$		
		A	B	C	A	B	C	A	B	C
Voltage	kV	53.5±0.01	69.5±0.01	69.6±0.01	69.6±0.01	53.5±0.01	69.5±0.01	69.5±0.01	69.6±0.01	53.5±0.01
	degree	−32.8±0.1	−149.5±0.1	89.4±0.1	−30.6±0.1	−152.8±0.1	90.5±0.1	−29.5±0.1	−150.6±0.1	87.2±0.1
Current	A	1412 ±8.47	9.7 ±0.06	9.7 ±0.06	9.6 ±0.06	1412 ±8.47	9.6 ±0.06	9.6 ±0.06	9.7 ±0.06	1412 ±8.47
	degree	−109.4 ±0.26	−60.6 ±0.26	179.0 ±0.26	63.7 ±0.26	130.7 ±0.26	176.2 ±0.26	60.9 ±0.26	−59.5 ±0.26	10.7 ±0.26
VT secondary winding voltage	V	$\begin{array}{l} 83\\ \pm 0.008 \end{array}$	$\begin{array}{l} 108\\ \pm 0.01 \end{array}$	$\begin{array}{l} 108\\ \pm 0.01 \end{array}$	$\begin{array}{l} 108\\ \pm 0.01 \end{array}$	$\begin{array}{l} 83\\ \pm 0.008 \end{array}$	$\begin{array}{l} 108\\ \pm 0.01 \end{array}$	$\begin{array}{l} 108\\ \pm 0.01 \end{array}$	$\begin{array}{l} 108\\ \pm 0.01 \end{array}$	$\begin{array}{l} 83\\ \pm 0.008 \end{array}$
	degree	−32.9 ±0.1	−149.6 ±0.1	89.3 ±0.1	−30.7 ±0.1	−152.9 ±0.1	90.4 ±0.1	−29.6 ±0.1	−150.7 ±0.1	87.1 ±0.1
CT secondary winding current	A	7.03 ±0.04	0.05 ±0.0003	0.05 ±0.0003	0.05 ±0.0003	7.03 ±0.04	0.05 ±0.0003	0.05 ±0.0003	0.05 ±0.0003	7.03 ±0.04
	degree	−108.9 ±0.26	−60.1 ±0.26	179.4 ±0.26	64.2 ±0.26	131.1 ±0.26	176.7 ±0.26	61.4 ±0.26	−59.0 ±0.26	11.1 ±0.26

**Table 2:** Models of filters for current and voltage symmetrical components.

Parameter	Sequence	Diagram
Voltage	Positive	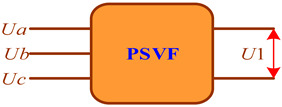
	Negative	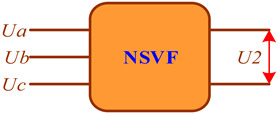
	Zero	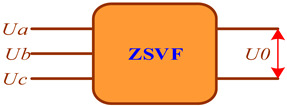
Current	Negative	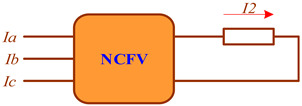
	Zero	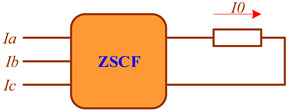

## Data Availability

The original contributions presented in the study are included in the article, further inquiries can be directed to the corresponding author.
